# Supplementary material for: Human-Induced Pluripotent Stem Cell-Derived Neural Progenitor Cells Showed Neuronal Differentiation, Neurite Extension, and Formation of Synaptic Structures in Rodent Ischemic Stroke Brains
Source: Cells. 2024 Apr 12;13(8):671. doi: 10.3390/cells13080671 (PMC11048851; doi:10.3390/cells13080671)
Supplement: Supplementary file 1 [file cells-13-00671-s001.zip › Supplementary Figures.pdf]

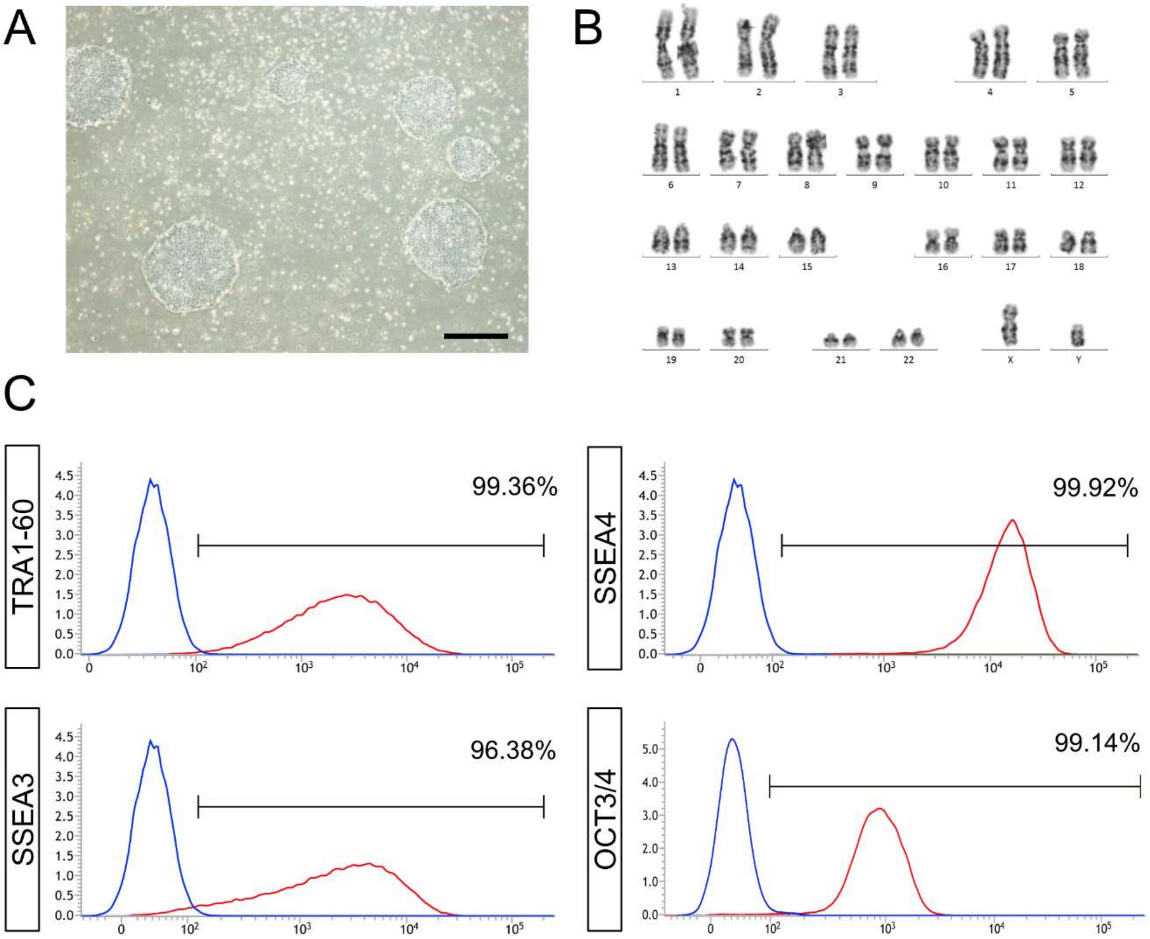

Supplementary Figure S1: Cellular properties of HLA-homo hiPSCs

A) Representative phase contrast image. Bar = 500 µm. B) Karyotype analysis carried out by G-banding. C) Flow cytometric analyses; red lines are the stained signal, and blue lines are negative controls.

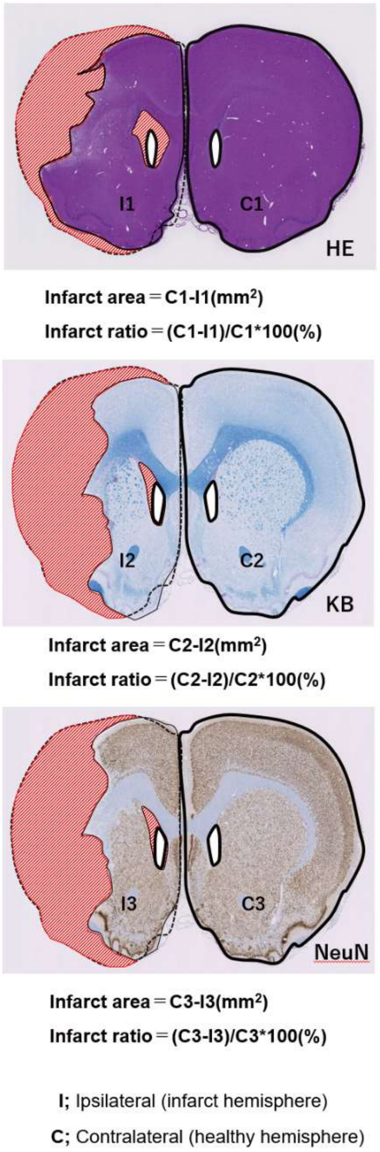

Supplementary Figure S2: Calculation method of infarct areas and ratios

Schematic diagram of the methods for calculating the infarct ratios using hematoxylin-eosin (HE), Klüver-Barrera (KB) staining, and NeuN immunohistochemical staining. I1 to 3 and C1 to 3 are HE-, KB-, and NeuN-positive areas (mm^2) on the ipsilateral infarct and contralateral healthy hemispheres, respectively. The infarct area, shown by red diagonal lines, was determined as the difference between C (1-3) and I (1-3). The infarct ratio was calculated as the ratio of the infarct area to C (1-3) and is shown as a percentage.

A

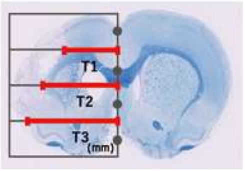

B

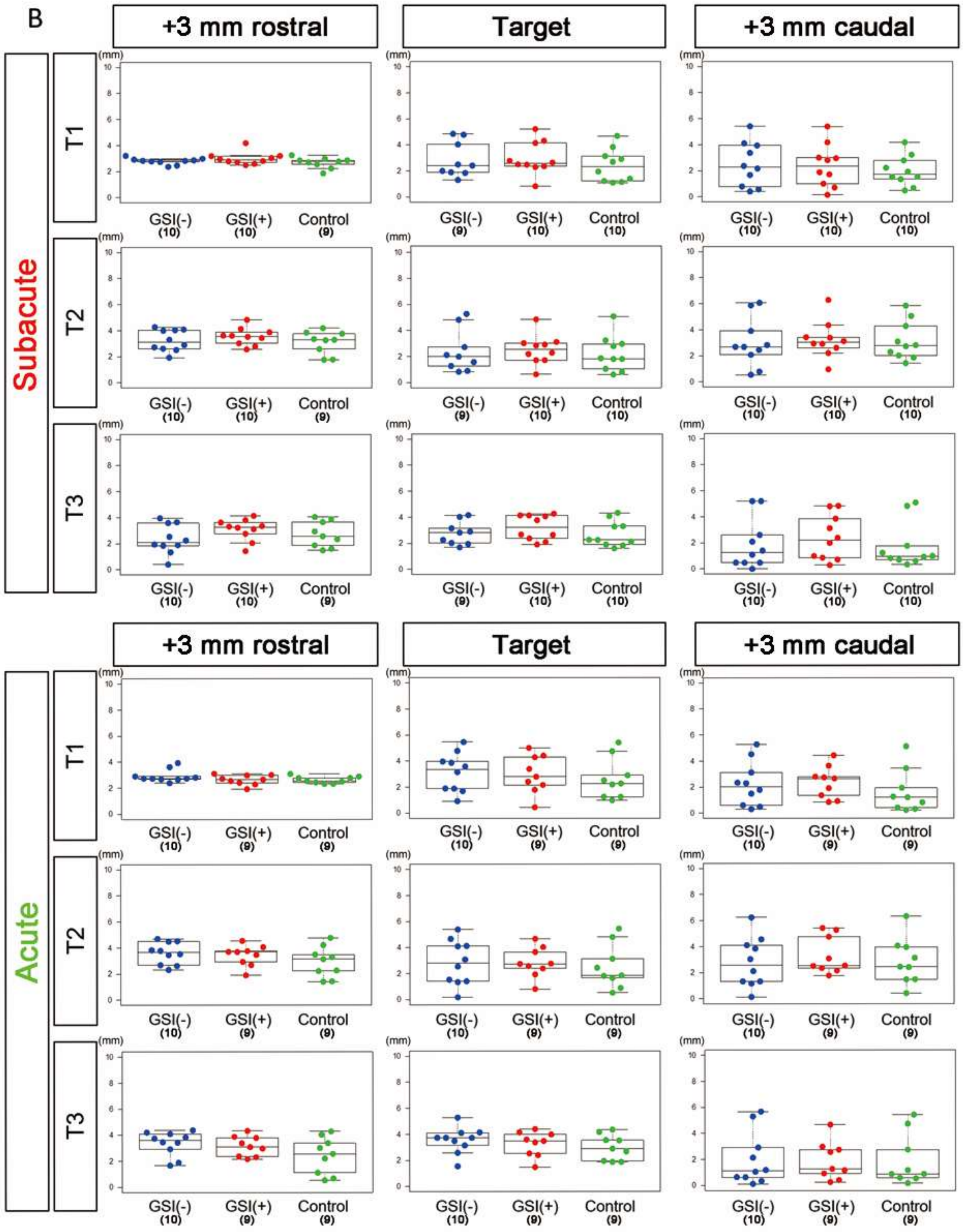

Supplementary Figure S3: Measurement method of residual brain tissue thickness

A) Schematic diagram of the methods for measuring the thickness of the remaining brain tissues. Thickness (mm) from the midline was measured at three points (T1, T2, and T3) in three Klüver-Barrera staining-coronal sections and shown in box-and-whisker plots: Target, the section centering the point of transplant; +3 mm rostral, +3 mm rostral sections from target; +3 mm caudal, +3 mm caudal sections from target. B) Results of residual brain tissue thickness in subacute or acute transplantation studies. Subacute: GSI (−) group (blue), GSI (+) group (red), control group (green). Acute: GSI (−) group (blue), GSI (+) group (red), control group (green).

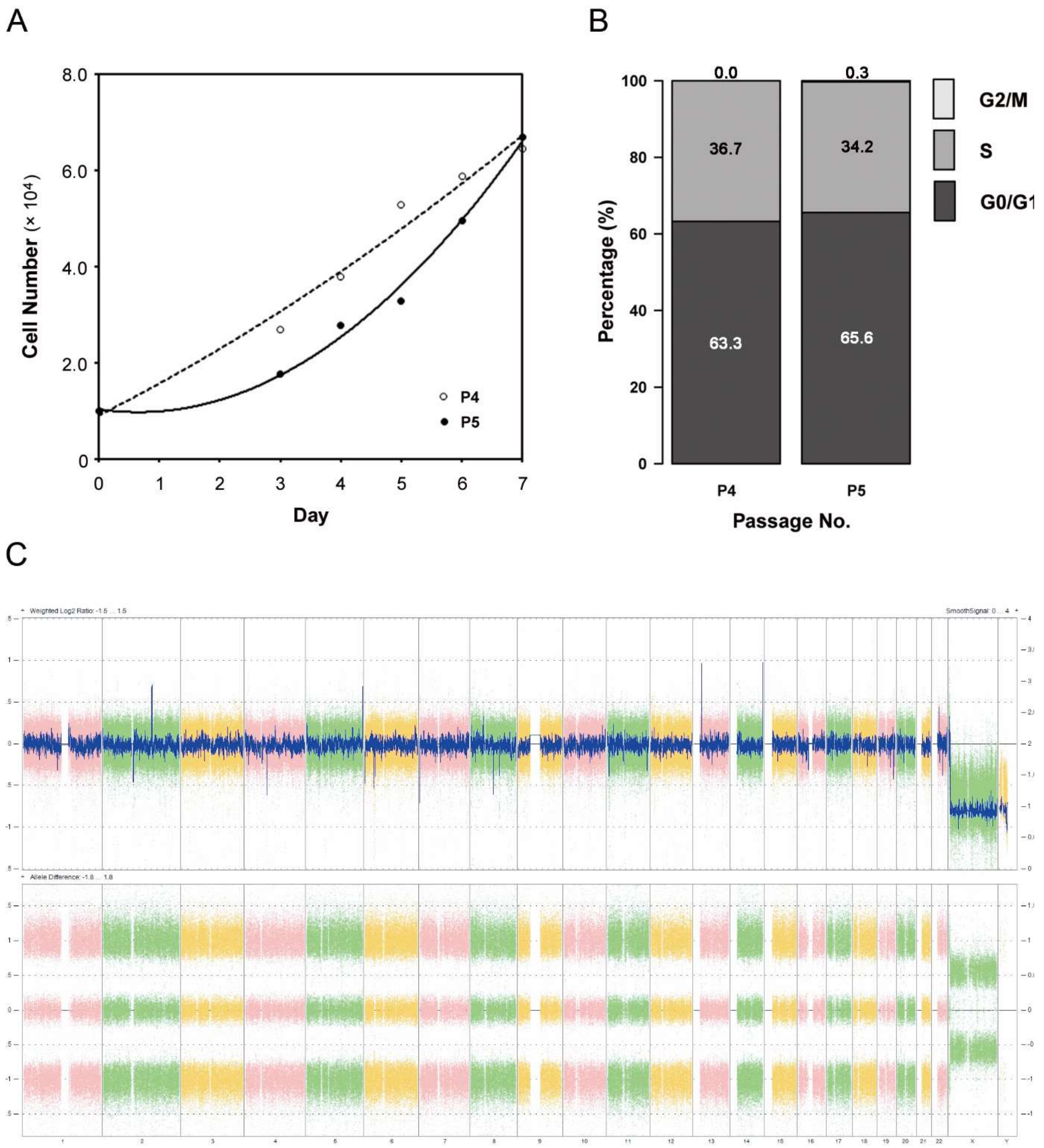

Supplementary Figure S4: Proliferation and genome properties of HLA-homo hiPSC-NPCs

A) Growth curves at passage 4 and passage 5 evaluated by measurement of ATP content in total viable cells. Cell number were determined from luminescence intensity (RLU). B) Cell cycle profiles at passage 4 and passage 5 using propidium iodide staining. C) Whole genome view of copy number analysis by the CytoScan HD Assay data, showing weighted log2 ratio (top panel) and allele difference (bottom panel).

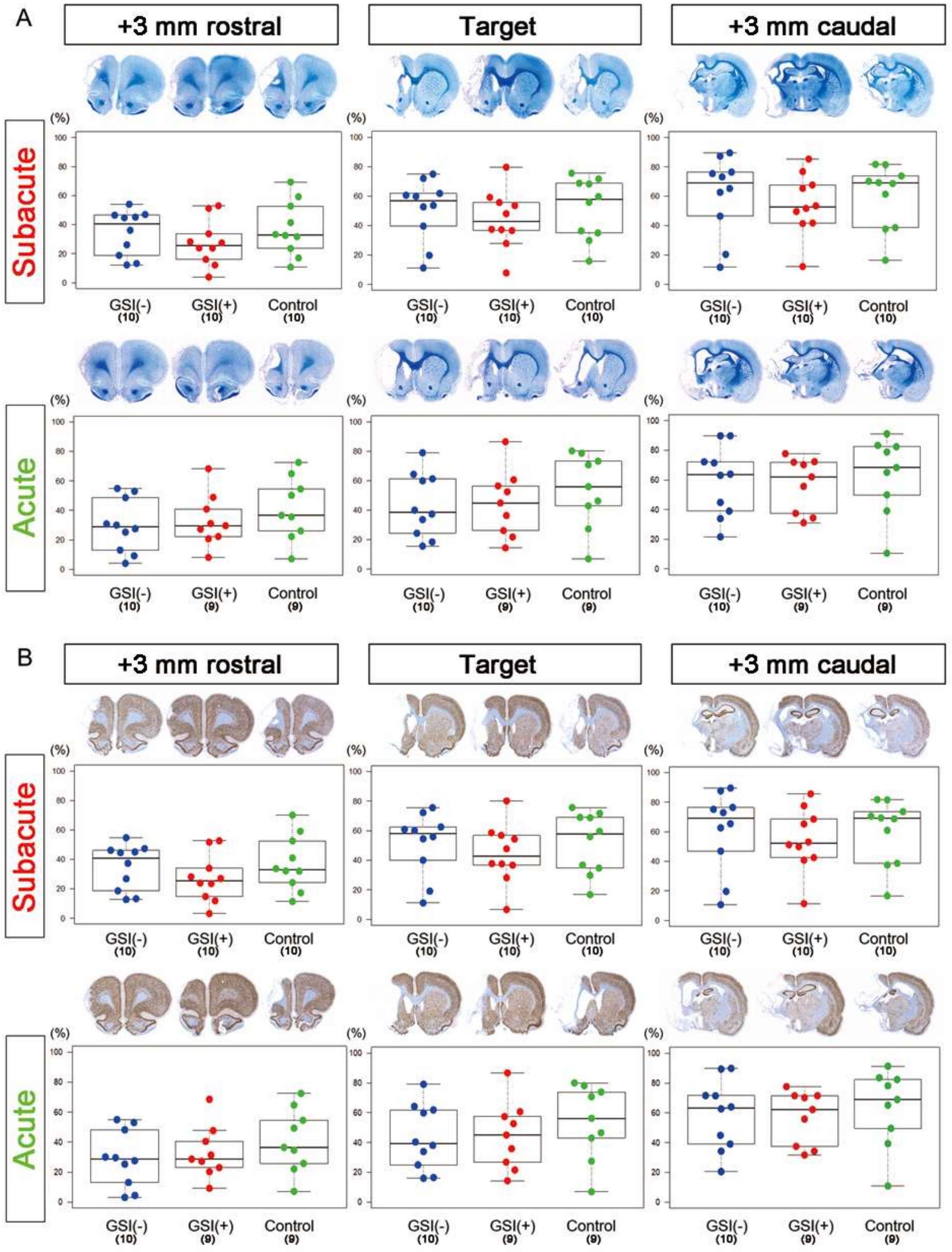

Supplementary Figure S5: Infarct ratios determined from Klüver-Barrera or NeuN immunohistochemistry staining in subacute and acute transplantation studies

The infarct ratio of each animal was measured using Klüver-Barrera staining (A) and NeuN immunohistochemistry (B) staining of coronal sections: target, the section centering the point of transplantation; +3 mm rostral, +3 mm rostral sections from the target; +3 mm caudal, +3 mm caudal sections from the target. The results are shown as box-and-whisker plots. Subacute: GSI (−) group (blue), GSI (+) group (red), and control group (green). Acute: GSI (−) group (blue), GSI (+) group (red), and control group (green).

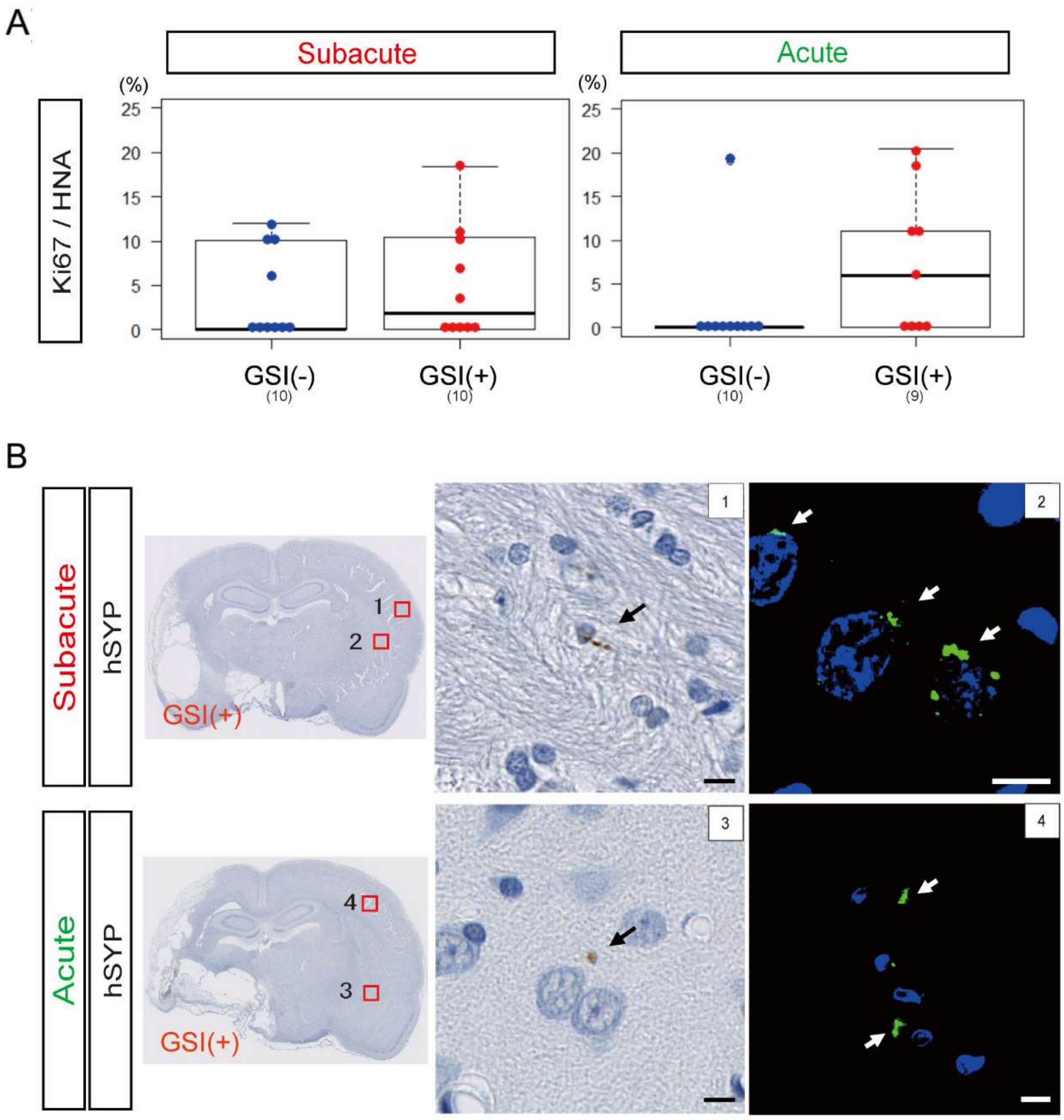

Supplementary Figure S6: Ratio of Ki-67/HNA positive cells and expression of human synaptophysin in contralateral healthy hemisphere

A) Ratios of Ki-67/NHA positive cells after 12 weeks from transplantation in GSI (−) and GSI (+) groups. Results are shown in box-and-whisker plots. B) Expression of hSYP in contralateral healthy hemisphere after 12 weeks from transplantation in GSI (−) and GSI (+) groups. Bar = 10 µm.

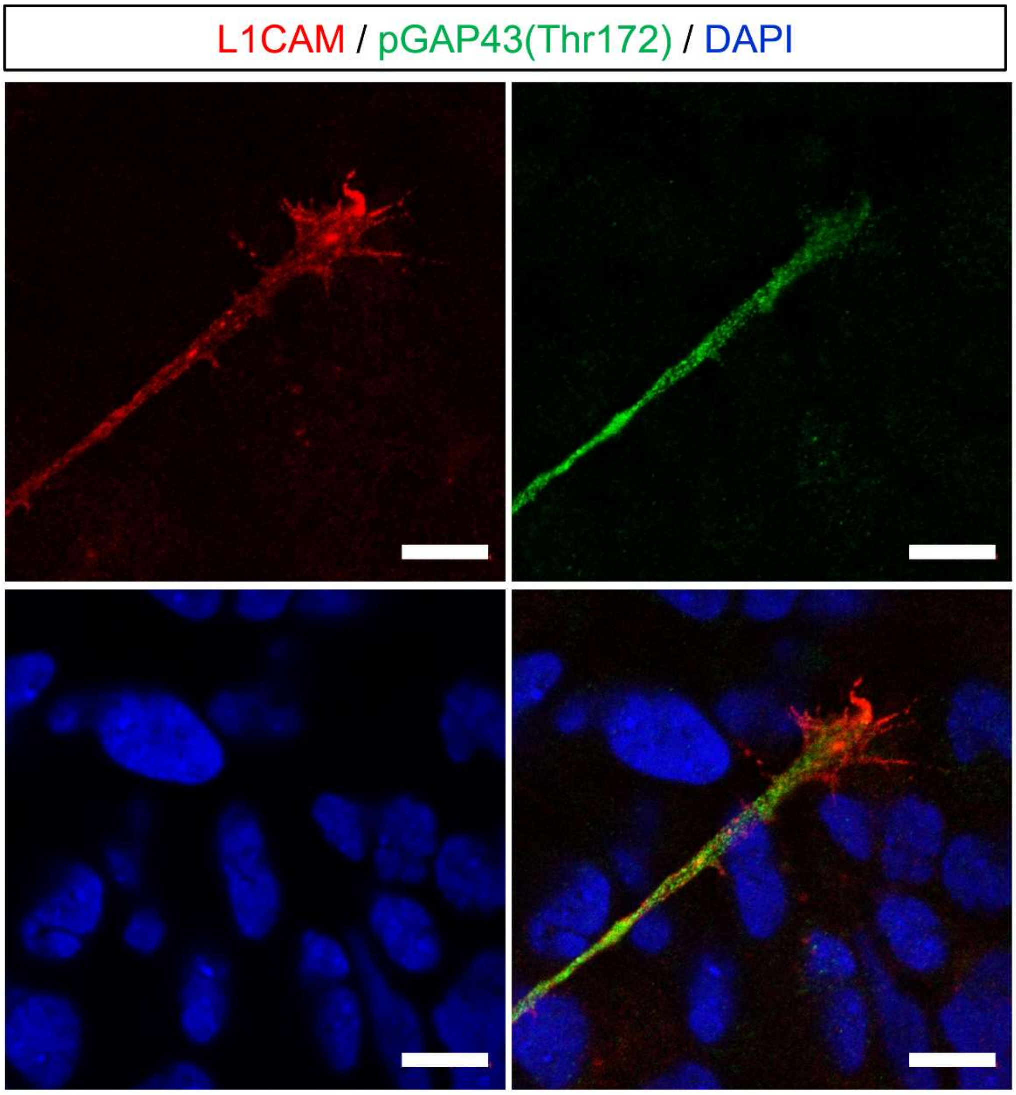

Supplementary Figure S7: Human neurite differentiated from hiPSC-NPCs.

Human neurites that extended from hiPSC-NPCs (1201C1 clone) after 3 days of in vitro differentiation were stained using both anti-L1CAM and anti-pGAP43(Thr172) antibodies. Bar = 10 µm.

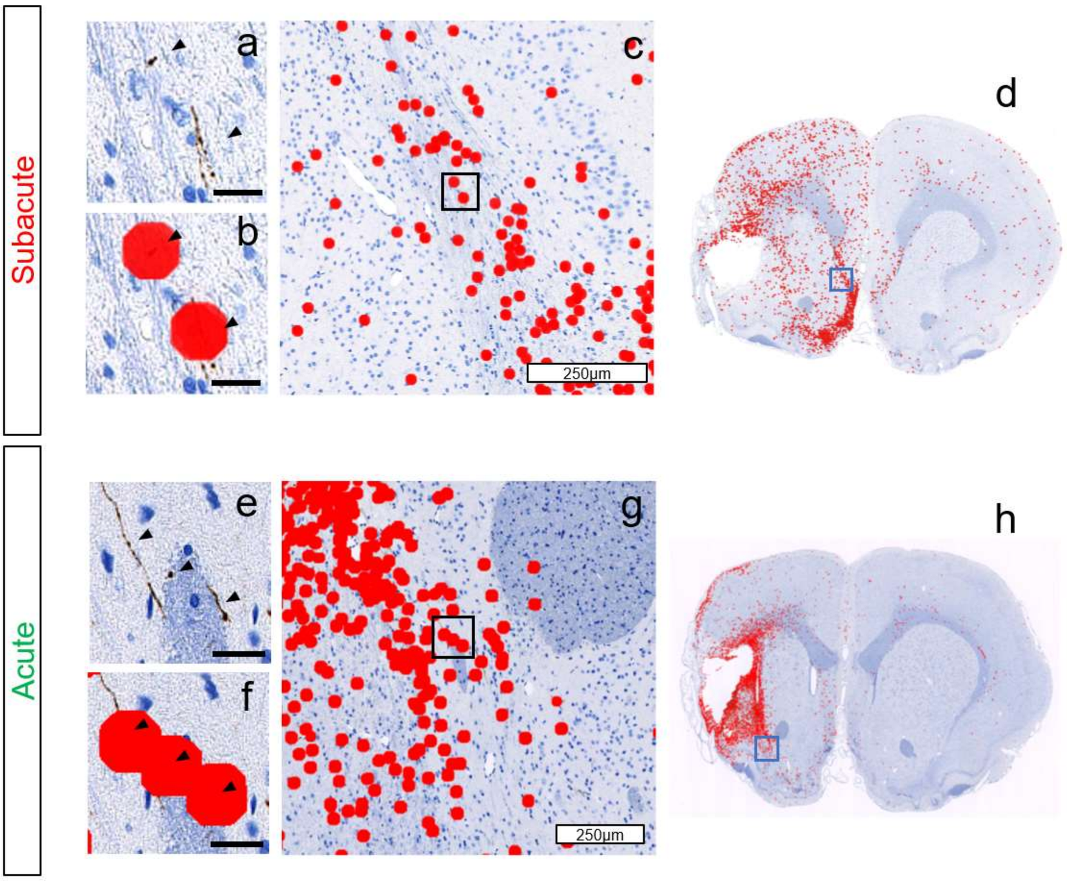

Supplementary Figure S8: Marking method of STEM121-positive fibers structures

The STEM121-positive portions (a and e) are marked by red dots (b and f) to visualize the distribution pattern of immunopositive regions (c, d, g, and h) (Figure 6C). Bar = 20 µm

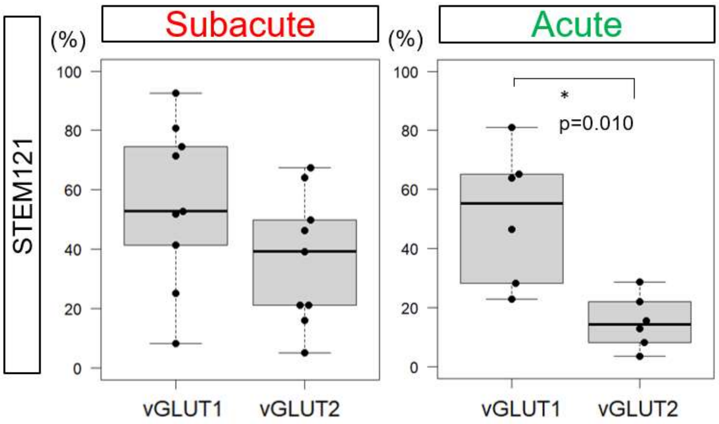

Supplementary Figure S9: Quantitative analysis of vGLUT1/2 expression

The percentage of double-positive areas of both STEM121 and vesicular glutamate transporter 1 (vGLUT1) or 2 (vGLUT2) was calculated in three regions of interest (ROIs) for each animal. Subacute (n = 3) or acute (n = 2)

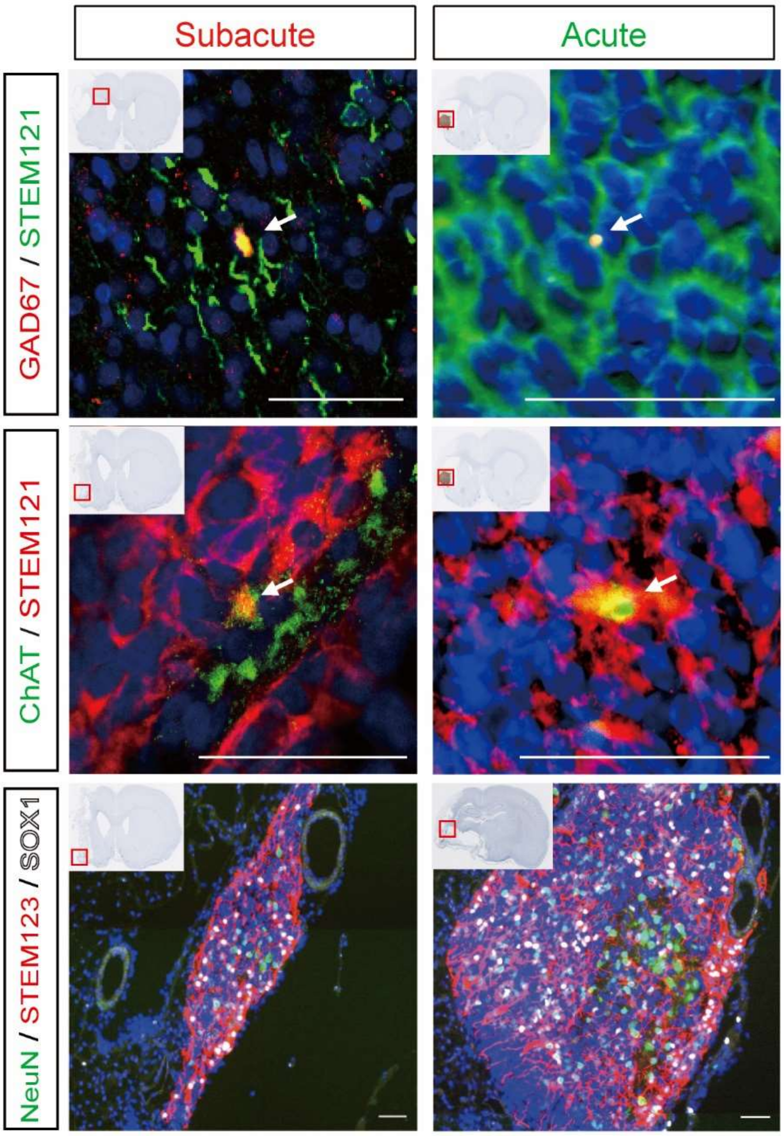

Supplementary Figure S10: Expression of neuronal and glial markers

The expression of GAD67 (inhibitory neurons) and choline acetyltransferase (ChAT, cholinergic neurons) in STEM121 positive cells, and human-specific GFAP (STEM123, astrocytes) was examined. Bar = 50 μm.

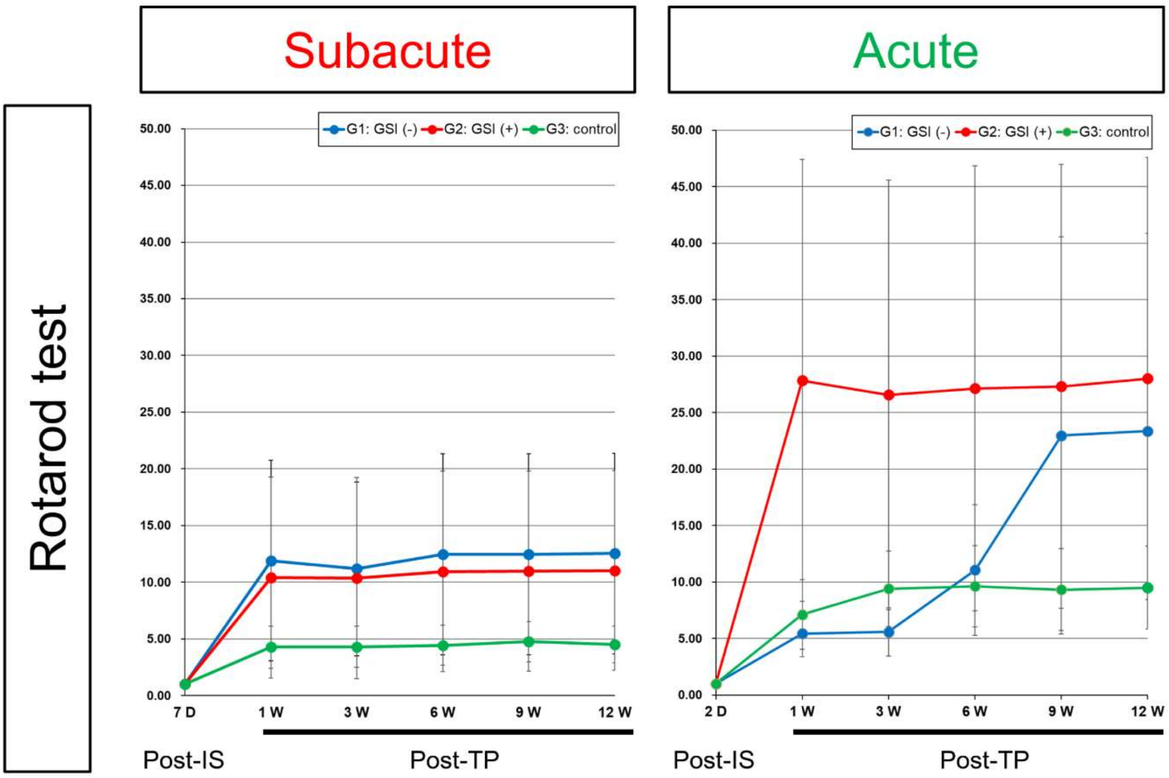

Supplementary Figure S11: Rotarod test

Results of rotarod test were calculated as relative ratio to baseline results [post-IS (ischemic) 2 days: acute transplantation, or 7 days: subacute transplantation)], and shown by mean ± SEM. Subacute: GSI (−) group (n = 10; blue), GSI (+) group (n = 10; red), and control group (n = 10; green). Acute: GSI (-) group (n = 10; blue), GSI (+) group (n = 9; red), and control group (n = 9; green).
