# Supplementary material for: Human-Induced Pluripotent Stem Cell-Derived Neural Progenitor Cells Showed Neuronal Differentiation, Neurite Extension, and Formation of Synaptic Structures in Rodent Ischemic Stroke Brains
Source: Cells. 2024 Apr 12;13(8):671. doi: 10.3390/cells13080671 (PMC11048851; doi:10.3390/cells13080671)
Supplement: Supplementary file 1 [file cells-13-00671-s001.zip › Supplementary Table S1.pdf]

**Supplementary Table S1: Primer Information**

| Official name | Symbol | Gene ID | MPN | Supplier |
|---|---|---|---|---|
| POU class 5 homeobox 1 | POU5F1 | 5460 | AIWR4JF | Thermo Fisher Scientific |
| SRY-box transcription factor 1 | SOX1 | 6656 | Hs01057642_s1 | Thermo Fisher Scientific |
| SRY-box transcription factor 2 | SOX2 | 6657 | Hs00602736_s1 | Thermo Fisher Scientific |
| paired box 6 | PAX6 | 5080 | Hs00240871_m1 | Thermo Fisher Scientific |
| nestin | NES | 10763 | Hs00707120_s1 | Thermo Fisher Scientific |
| glyceraldehyde-3-phosphate dehydrogenase | GAPDH | 2597 | Hs99999905_m1 | Thermo Fisher Scientific |
