# Supplementary material for: Human-Induced Pluripotent Stem Cell-Derived Neural Progenitor Cells Showed Neuronal Differentiation, Neurite Extension, and Formation of Synaptic Structures in Rodent Ischemic Stroke Brains
Source: Cells. 2024 Apr 12;13(8):671. doi: 10.3390/cells13080671 (PMC11048851; doi:10.3390/cells13080671)
Supplement: Supplementary file 1 [file cells-13-00671-s001.zip › Supplementary Table S2.pdf]

Supplementary Table S2: List of primary antibodies used

Antigen		Host Species, Clonality	Clone	MPN	Supplier	Application
PSA-NCAM	Polysialic acid-NCAM	M, mono	2-2B	MAB5324	Merck	FCM
GD2	Ganglioside GD2	M, mono	14.G2a	554272	BD	FCM
SOX1	SRY-Related HMG-Box Gene 1	M, mono	N23-844	561592	BD	FCM
SOX1	SRY-Related HMG-Box Gene 1	M, mono	N23-844	560749	BD	ICC
SOX1	SRY-Related HMG-Box Gene 1	G, poly	-	AF3369	R&D	IHC-F
TRA1-60	-	M, mono	TRA1-60	560071	BD	FCM
TRA1-60	-	M, mono	TRA1-60	560173	BD	FCM
SSEA3	Stage specific embryonic antigen 3	R, mono	MC-631	MAB4303-I	Merck	FCM
SSEA4	Stage specific embryonic antigen 4	M, mono	MC813-70	560126	BD	FCM
OCT3/4	Octamer-binding transcription factor 3/4	M, mono	40/Oct-3	560186	BD	FCM
OCT3/4	Octamer-binding transcription factor 3/4	M, mono	40/Oct-3	611203	BD	ICC
NES	Nestin	M, mono	10C2	MAB5326	Merck	ICC
βIII-tubulin	Class III β-tubulin	M, mono	TUJ1	801201	BioLegend	ICC
GFAP	Glial fibrillary acidic protein	Rb, mono	EP672Y	ab33922	Abcam	ICC
NeuN	Neuron-Specific Nuclear Protein	Rb, mono	EPR12763	ab177487	Abcam	IHC-F
NeuN	Neuron-Specific Nuclear Protein	M, mono	A60	MAB377	Merck	IHC-C
HNA	Human nuclear antigen	M, mono	3E1.3	MAB4383	Merck	IHC-C, IHC-F
Ki-67	-	M, mono	MIB-1	M7240	Agilent Technologies	IHC-C
ELAVL3/4 [HuC/D]	Embryonic lethal abnormal vision-like 3/4	Rb, mono	EPR19098	ab184267	Abcam	IHC-F
ELAVL3/4 [HuC/D]	Embryonic lethal abnormal vision-like 3/4	M, mono	16A11	A21271	Thermo Fisher Scientific	ICC
hSYP	Human synaptophysin	M, mono	EP10	ADI-905-782	Enzo Life Sciences	IHC-C, IHC-F
STEM121	Human cytoplasmic marker	M, mono	STEM121	Y40410	Takara Bio	IHC-C, IHC-F
STEM123	Human Glial fibrillary acidic protein	M, mono	STEM123	Y40420	Takara Bio	IHC-F
SOX10	SRY-Related HMG-Box Gene 10	Rb, mono	EPR4007-104	ab180862	Abcam	IHC-F
DreA	DrebrinA	Rb, poly	-	28023	IBL	IHC-F
vGLUT1	Vesicular glutamate transporter 1	Rb, poly	-	135 303	Synaptic systems	IHC-F
vGLUT2	Vesicular glutamate transporter 2	Rb, poly	-	135 403	Synaptic systems	IHC-F
ChAT	Choline acetyltransferase	G, poly	-	AB144P	Merck	IHC-F
GAD67	Glutamate decarboxylase 67	M, mono	1G10.2	MAB5406	Merck	IHC-F
p-GAP43 (Thr 172)	Phosphorylated growth associated protein 43	M, mono	19-9A	017-25411	Fujifilm Wako Chemical	ICC, IHC-F
HLA-ABC-Biotin	Human leukocyte antigen-ABC	M, mono	EMR8-5	AB-46-B	Hokudo	IHC-F
L1CAM	L1 cell adhesion molecule	Rb, mono	EPR23241-224	ab270455	Abcam	ICC

M, mouse; R, rat; Rb, rabbit; G, goat; mono, monoclonal; poly, polyclonal; FCM, flow cytometry; ICC, immunocytochemical analysis (in vitro); IHC-C, immunohistochemical analysis (chromogenic detection in vivo); IHC-F, immunohistochemical analysis (fluorescent detection in vivo)
