# Supplementary material for: Human-Induced Pluripotent Stem Cell-Derived Neural Progenitor Cells Showed Neuronal Differentiation, Neurite Extension, and Formation of Synaptic Structures in Rodent Ischemic Stroke Brains
Source: Cells. 2024 Apr 12;13(8):671. doi: 10.3390/cells13080671 (PMC11048851; doi:10.3390/cells13080671)
Supplement: Supplementary file 1 [file cells-13-00671-s001.zip › Supplementary Table S3.pdf]

Supplementary Table S3: Neurological Deficit Score

	Score
<u>Forelimb flexion</u>	
Animals were held gently by the tail, suspended about 10 cm above the floor, and observed for forelimb flexion.	
· animals extend both forelimbs toward the floor	0
· there is a slight difference between forelimb extension	1
· there is about 90-degree flexion in the affected forelimb	2
· impossible to move the affected forelimb	3
<u>Hindlimb flexion</u>	
The animal's head was covered with a hand, and under a sedative condition the hindlimbs were gently pulled toward the tail, and observed retractive power.	
· retractive power is not different between hindlimbs	0
· retractive power of the right hindlimbs weaker than that of the left hindlimb	1
· the right hindlimb is extended abnormally, and is retractable when the sole is touched with a finger	2
· the right hindlimb is extended abnormally, and is not retractable when the sole is touched with a finger	3
<u>Rotational behavior,</u>	
Animals were held gently by the tail, and with their forelimbs on the floor, and observed rotational behavior.	
· animal walks ahead, and can walk to the right and left	0
· animal usually walks ahead, and cannot walk towards the left	1
· animal usually walks towards the right, and can walk ahead	2
· animal walks towards the right, and cannot walk ahead	3
<u>Lateral displacement</u>	
On stable condition, gentle lateral pressure was applied for the animal's body, and observed resistance to a lateral push.	
· animal resisted sliding equally in both directions	0
· resistance to a lateral push towards the right is slightly reduced, but keep the posture	1
· resistance to a lateral push towards the right is markedly reduced, and difficult to keep the hindlimb	2
· resistance to a lateral push towards the right is markedly reduced and the animal falls on its back	3
<u>General posture</u>	
The general posture of each animal was observed.	
· animal's posture after the operation is not different from that in normal animals	0
· when looking at animals from the back, the left forelimb and hindlimb can be seen, because balance has shifted to the right	1
· when looking at animals from the back, the left forelimb and hindlimb can be seen, and when looking at animals from the front, their bodies are leaning slightly	2
· when looking at animals from the back, the left forelimb and hindlimb can be seen, and when looking at animals from the front, their bodies are leaning markedly	3
Total score	(0-15)
